# Transcutaneous auricular vagus nerve stimulation alleviates inflammation-induced depression by modulating peripheral-central inflammatory cytokines and the NF-κB pathway in rats

**DOI:** 10.3389/fimmu.2025.1536056

**Published:** 2025-05-16

**Authors:** Xingke Song, Haohan Zhu, Zijie Chen, Yifei Wang, Jinling Zhang, Yu Wang, Peijing Rong, Junying Wang

**Affiliations:** ^1^ Institute of Acupuncture and Moxibustion, China Academy of Chinese Medical Sciences, Beijing, China; ^2^ Institute Of Basic Research In Clinical Medicine, China Academy of Chinese Medical Sciences, Beijing, China

**Keywords:** transcutaneous auricular vagus nerve stimulation, inflammation, NF-κB, LPS, depression

## Abstract

**Objectives:**

This study aims to investigate the mechanisms of transcutaneous auricular vagus nerve stimulation (taVNS) in a lipopolysaccharide (LPS)-induced acute inflammatory depression model in rats, focusing on the regulation of peripheral pro- and anti-inflammatory cytokines and the effects on the NF-κB signaling pathway in the prefrontal cortex.

**Methods:**

A depressive-like behavior model was established via intraperitoneal injection of LPS, and rats were randomly assigned to a normal group, a model group, and a taVNS intervention group. Behavioral assessments included the sucrose preference test (SPT), open field test (OFT), and elevated plus maze test (EPM) to evaluate depressive-like behaviors. Bio-Plex suspension array technology was used to detect inflammatory cytokine levels in serum, and Western blotting was performed to analyze the expression of NF-κB signaling pathway-related proteins in the prefrontal cortex.

**Results:**

Behavioral tests demonstrated that LPS-induced rats exhibited significant depressive-like behaviors, including reduced sucrose preference, decreased activity levels in the open field, and restricted exploratory behavior in the elevated plus maze. taVNS intervention significantly alleviated these depressive-like behaviors. Serum analysis revealed that pro-inflammatory cytokines (e.g., IL-1β, TNF-α, MCP-1, IL-18, MIP-1α, and MIP-3α) were markedly elevated, while anti-inflammatory cytokines IL-4 and IL-10 were significantly reduced in the model group. taVNS intervention reversed these changes. Western blot analysis showed significant activation of the NF-κB signaling pathway in the model group, characterized by increased p-P65/P65 and p-IκB/IκB ratios and elevated TNF-α expression. taVNS intervention suppressed excessive activation of the NF-κB pathway by reducing p-P65 and TNF-α levels and stabilizing IκB expression.

**Conclusions:**

This study demonstrates that taVNS significantly improves LPS-induced depressive-like behaviors by modulating peripheral pro- and anti-inflammatory cytokine levels and inhibiting the activation of the NF-κB signaling pathway in the prefrontal cortex. These findings highlight the critical role of taVNS in the regulation of the peripheral-central inflammation network and provide theoretical support for the development of taVNS-based non-invasive neuromodulation therapies.

## 1 Introduction

Depression is a prevalent and debilitating mental disorder that imposes a significant burden on individuals and healthcare systems globally (1). While traditionally linked to neurotransmitter imbalances, emerging evidence has highlighted the crucial role of inflammation in the pathophysiology of depression (2, 3).Recent studies have demonstrated that peripheral inflammation plays a critical role in the development of depressive symptoms within the central nervous system (CNS). Peripheral pro-inflammatory cytokines, such as IL-6, TNF-α, and IL-1β, can cross the blood-brain barrier, activate microglia, and induce neuroinflammation (4). This process disrupts neurotransmitter metabolism and synaptic plasticity, ultimately contributing to the onset of depressive symptoms (4). Additionally, peripheral inflammation can transmit signals to the CNS via pathways such as the vagus nerve, directly affecting brain regions associated with emotional regulation, including the hippocampus and prefrontal cortex (5, 6).This systemic-to-central signaling, often termed the “peripheral-central inflammation axis,” has been implicated in the onset and progression of depressive symptoms (7). In this context, the LPS-induced depression model has become a crucial foundation for investigating how peripheral inflammation contributes to central depressive states (3, 8). LPS, a component of the cell walls of gram-negative bacteria, triggers a strong immune response by activating Toll-like receptor 4 (TLR4) on immune cells, leading to the release of pro-inflammatory cytokine (9). These cytokines not only mediate peripheral inflammation but also impact the central nervous system by disrupting neurochemical homeostasis, altering synaptic plasticity, and inducing neuroinflammation, ultimately leading to depression (10).Among the central pathways influenced by inflammation, the nuclear factor kappa B (NF-κB) signaling cascade is a pivotal regulator of neuroimmune responses and is closely associated with depressive behaviors (11).

taVNS has emerged as a promising non-invasive neuromodulation therapy with anti-inflammatory and antidepressant potential. Several clinical evidence have demonstrated the efficacy of taVNS for depression (12, 13).In terms of anti-inflammatory and antidepressant effects, studies have shown that taVNS significantly alleviates depressive symptoms by modulating peripheral immunity and neuroinflammatory responses in the CNS. Our previous studies showed that taVNS inhibited microglial activation in the hippocampus, hypothalamus, exerting anti-inflammatory effects through the α7nAChR/NF-κB/IL-1β signaling pathway for the CUMS induced depressed rats (14), and the expression of TNF-α in the hypothalamus and amygdala for the LPS induced depressed rats (15). Furthermore, It was also showed that taVNS regulate the α7nAChR/JAK2/STAT3 signaling pathway in the spleen, modulating serum levels of IL-10 and chemokine ligand 1, and upregulating p-JAK2 and p-STAT3 protein expression in the spleen, thereby improving LPS-induced depressive-like behaviors in rats (16).Other studies have also reviewed how taVNS exerts its antidepressant effects through anti-inflammatory actions in both peripheral and central systems (17).These findings suggest that the coordinated regulation of peripheral immunity and the CNS by taVNS plays a crucial role in its antidepressant effects.

Despite the progress achieved, the central regulatory mechanisms in the prefrontal cortex and the peripheral anti-inflammatory effects of taVNS in LPS-induced acute inflammatory depression models remain to be further elucidated. This study aims to investigate the peripheral and central regulatory effects of taVNS by assessing changes in pro-inflammatory and anti-inflammatory cytokines in peripheral blood, as well as key molecular components of the NF-κB signaling pathway in the prefrontal cortex.These findings will provide further insights into the mechanisms of taVNS in treating inflammation-related depression and contribute to the development of auricular-based neuromodulation therapies for depression.

## 2 Materials and methods

### 2.1 Animals and grouping

Eighteen healthy 8-week-old male Sprague-Dawley (SD) rats (SPF grade) weighing 180–200 g were obtained from Beijing SPF Biotechnology Co., Ltd. (Production License Number: SCXK (Beijing) 2019-0010). All animals were housed under controlled conditions: room temperature of 22 ± 2°C, relative humidity of 55 ± 5%, and a 12-hour light/dark cycle (lights on at 7:00 AM and off at 7:00 PM). Food and water were provided ad libitum.

In order to reduce the stress response, the rats were adaptively fed for one week. The rats were randomly divided into three groups using a random number table: a normal group, a model group, and a taVNS group, with six rats in each group.

All experimental procedures were approved by the Animal Ethics Committee of the Institute of Acupuncture and Moxibustion, China Academy of Chinese Medical Sciences (Approval Number: D2019-02-11-3), and were conducted in accordance with the Guide for the Care and Use of Laboratory Animals.

### 2.2 Model establishment

A depression-like behavior rat model was established via intraperitoneal injection of lipopolysaccharide (Product No. S11060, Shanghai Yuanye Bio-Technology Co., Ltd.). According to previous studies, 24 hours post-injection is the optimal time point for observing depression-like behaviors (17, 18).

On the afternoon of day 6 (4:00 PM), rats in the model group and the taVNS group received intraperitoneal injections of LPS at a dose of 1 mg/kg, diluted in 0.9% sodium chloride solution. Rats in the normal group were injected with an equivalent volume of 0.9% sodium chloride solution.

### 2.3 TaVNS intervention method

**Figure 1:**
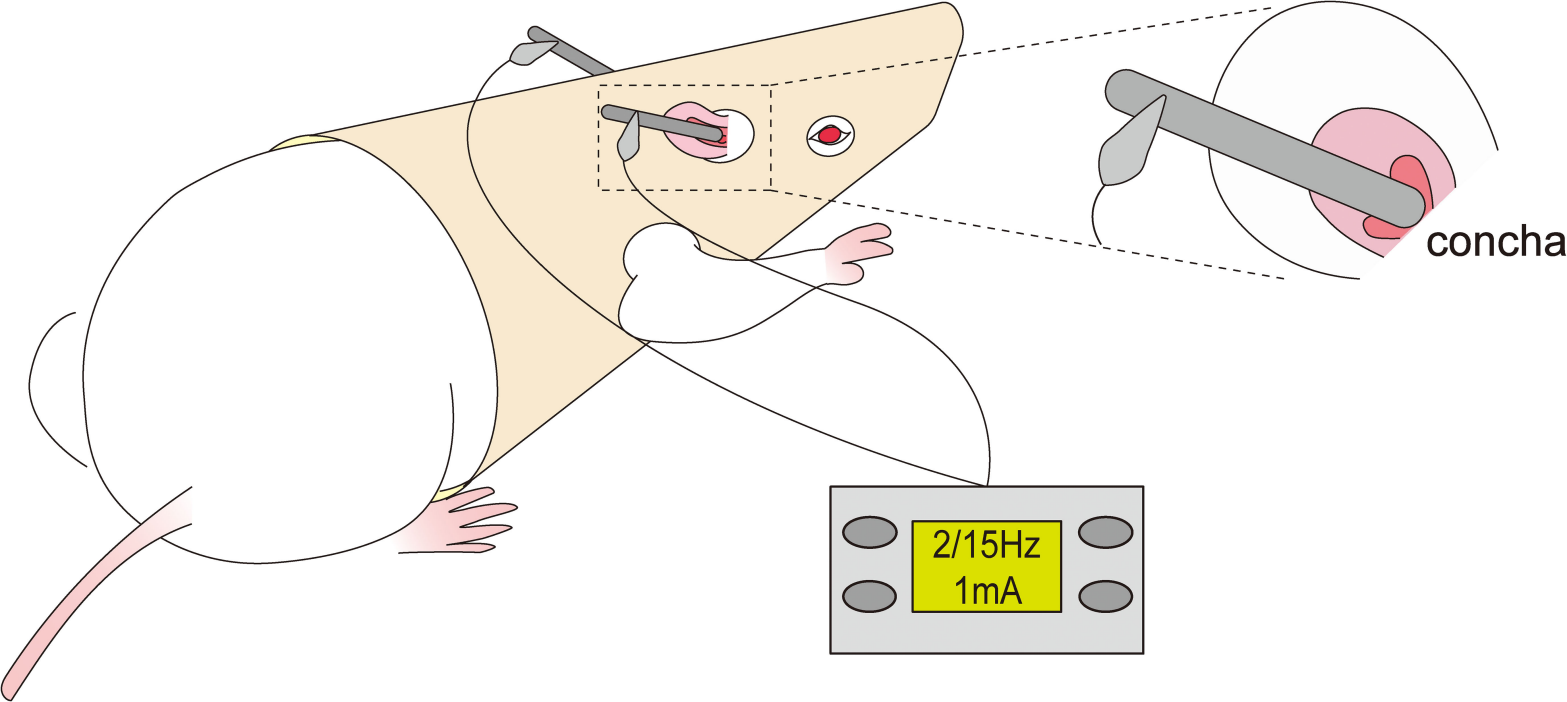
Schematic of transcutaneous auricular vagus nerve stimulation.

Conscious and free-moving rats in the taVNS group were administrated taVNS treatment for consecutive 7 days. The rats were put on the rats’ jacket exposing the ears and forelimbs which was designed by ourselves and got a patent for practical novelty (ZL 201921389869.4).Then, the stimulation electrode was placed in the ear concha of rats to ensure effective current transmission(**Figure 1**).

**Figure 2:**
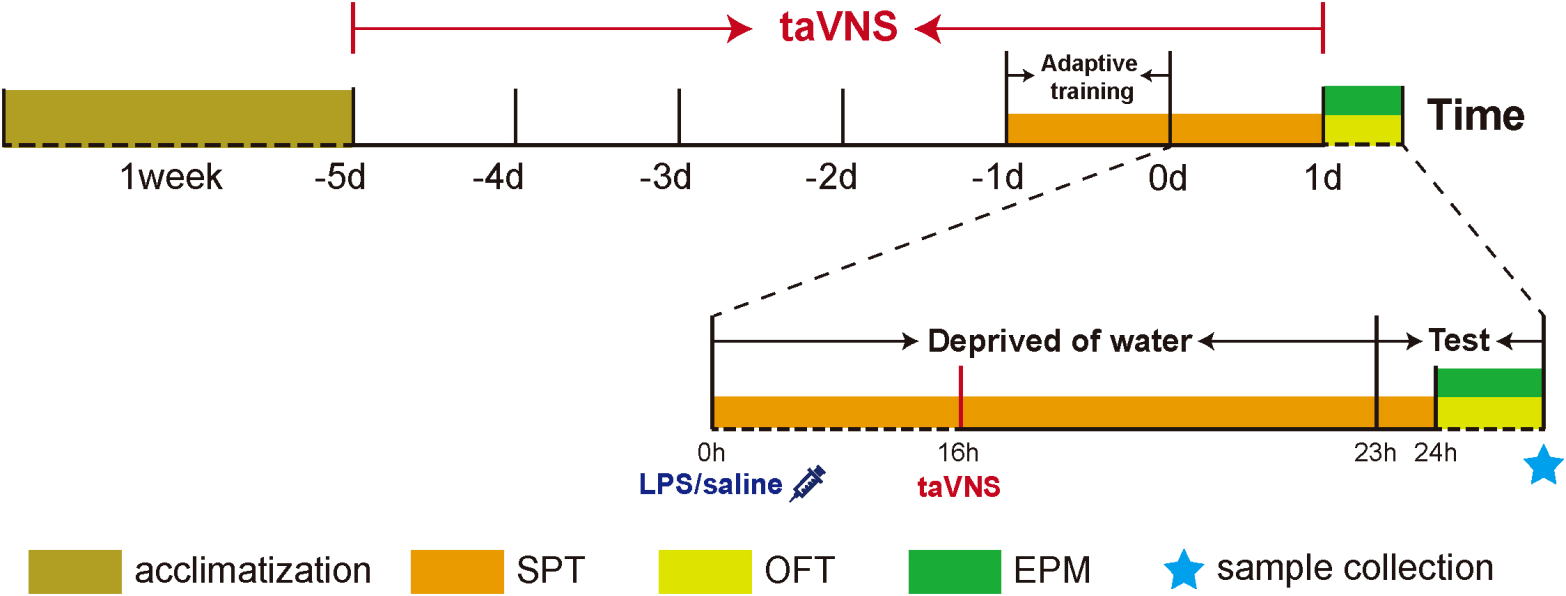
Description of experimental design. Note: SPT, Sucrose Preference Test; OFT, Open Field Test; EPM, Elevated Plus Maze Test; taVNS,Transcutaneous Auricular Vagus Nerve Stimulation.

In order to ensure the effective intervention of taVNS for depressive behavior,the intervention protocol was as follows: the rats were treated with taVNS once daily 7 days between 8:00 AM and 10:00 AM to weaken the influence of biological rhythm(**Figure 2**). After adapting to the electrodes and the rats’ jacket for 15min, the rats were given electrical stimulation. The stimulation parameters were set to sparse-dense waves at a frequency of 2 Hz/15 Hz, with a current intensity of 1 mA, for 30 minutes per session, using a Hans100A electroacupuncture device (Nanjing Jisheng Medical Technology Co., Ltd., China).

### 2.4 Sucrose preference test

The percentage of sucrose solution consumption is an important objective indicator for assessing anhedonia (19). The procedure was conducted according to our previous study (15). The rats were isolated in their own cage before the adaptive training.Rats were subjected to adaptive training with pure water and 1% sucrose solution for 24 hours. Following this, the rats were deprived of water for 23 hours. In the subsequent 1 hour period, they were given the choice to drink either pure water or 1% sucrose solution.The sucrose preference rate was calculated using the following formula: Sucrose Preference Rate = Sucrose Solution Consumption/(Sucrose Solution Consumption+Pure Water Consumption)×100% (20).

### 2.5 Open field test

The rats were acclimatized for 7 days prior to the experiment to enable their familiarization to the investigator. One hour before testing, the rats were placed in the testing room for acclimatization to the room. There should be appropriate lighting above the open field box to avoid shadows and prevent the rats from feeling uncomfortable. The video camera is placed into the clamp of the retort stand right above the open field box in order to allow complete coverage of the box.The behavioural testing room is a quiet zone to ensure that the test rats is not disturbed and can still be observed by the investigator. The experiment was conducted in a quiet environment. The OFT was conducted after the taVNS intervention was completed. The open field apparatus consisted of a black plastic box with dimensions of 100 cm × 100 cm × 40 cm, with the interior divided into 25 equal squares (5 × 5) by white lines. At the start of the test, each rat was gently placed in the center of the box and allowed to explore freely for 5 minutes. The activity of the rats was recorded and analyzed using the video-based behavioral analysis system(JLBehv-LAR-1,Shanghai Jiliang Software Technology Co., LTD, Shanghai, China).

The parameters recorded included the total and central duration of activity. After each rat’s test, the bottom of the box was wiped with 75% alcohol, and any feces or other residues were promptly removed to maintain a clean experimental environment.

### 2.6 Elevated plus maze test

The rats were acclimatized to the holding facility and the investigator at least 7 days prior to the experiment. One hour before the test, the rats were placed in the behavioral testing room. There should be appropriate lighting above the elevated plus maze to avoid shadows and prevent the rats from feeling uncomfortable. The elevated plus maze system(DigBehv-EPMG, Shanghai Jilang Software Technology Co., LTD, Shanghai, China) is placed in a separate compartment enclosed by a cloth curtain to help minimize stresses. The camera is mounted on the retort stand so that it is positioned above the elevated plus maze. Low ambient noise should be maintained throughout behavioral testing. Each rat was placed on the central platform of the elevated plus maze, facing an open arm. The rat’s activity was observed and recorded for 5 minutes, focusing on the time spent in the open arms and the number of entries into the open arms. After each test, the platform was cleaned with 75% alcohol and a damp cloth, and dried thoroughly to remove odors and prevent interference with the behavior of subsequent test animals.

### 2.7 Western blot

Rats in each group were anesthetized via intraperitoneal injection of 1% sodium pentobarbital (40 mg/kg) and subsequently euthanized. Tissue from the prefrontal cortex were collected to detect the expression levels of p-P65, P65, p-IκB, and IκB proteins. The tissues were homogenized in RIPA lysis buffer, and total protein was extracted and quantified using the BCA assay.

Protein samples were separated via gel electrophoresis and transferred onto PVDF membranes. The membranes were blocked with TBST containing 5% non-fat milk for 1 hour at room temperature. After blocking, the membranes were incubated overnight at 4°C with the following primary antibodies: rabbit anti-p-P65 (CST, 3033, 1:1000), rabbit anti-P65 (CST, 8242, 1:2000), rabbit anti-p-IκB (CST, 2859, 1:5000), mouse anti-IκB (CST, 4814, 1:2000), rabbit anti-TNF-α(abcam,ab66579,1:1000) and β-actin (Immunoway, YM3028, 1:5000).

After washing, the membranes were incubated with HRP-conjugated secondary antibodies (1:500) for 2 hours at room temperature. Protein bands were visualized via film exposure and analyzed using Image-Pro Plus software. The grayscale value of the target protein bands was normalized to that of β-actin, which served as the internal control. The ratio of the target protein’s grayscale value to that of β-actin was calculated as the relative expression level of the target protein.

### 2.8 Bio-plex suspension array technology

Rats in each group were anesthetized via intraperitoneal injection of 1% sodium pentobarbital (40 mg/kg). Blood was collected from the abdominal aorta into EDTA-containing anticoagulant tubes and allowed to stand for 20 minutes. The blood samples were then centrifuged at 3500 r/min for 15 minutes at 4°C, and the plasma was collected and stored at -80°C until further analysis.

Before testing, plasma samples were thawed on ice at 4°C and centrifuged again. The supernatant was collected for the experiment. According to the instructions of the Bio-Plex assay kit (12005641, Bio-Rad Laboratories, California, USA), standard samples were diluted to prepare a gradient standard curve, and microspheres were diluted accordingly.

In a 96-well plate, diluted microspheres, standards, and plasma samples were sequentially added. Detection antibodies and diluted Streptavidin-PE were then added step by step, and the samples were incubated under light-protected conditions. The wells were washed according to the protocol. After adding the incubation buffer, the samples were vortexed thoroughly and analyzed using a Bio-Plex instrument(Bio-Plex 200,Bio-Rad Laboratories, California, USA). The concentrations of target cytokines, including TNF-α, IL-1β, MCP-1, IL-18, MIP-1α, MIP-3α, IL-4, and IL-10 were calculated based on the standard curve.

### 2.9 Statistical analysis

Statistical analyses were performed using GraphPad Prism 8 software. Measurement data conforming to a normal distribution were expressed as mean ± standard deviation (Mean ± SD). Group comparisons for Western blot,Bio-Plex,the open field test, elevated plus maze test, and sucrose preference test results were conducted using one-way analysis of variance (ANOVA).

For data with homogeneous variances, *post hoc* analyses were performed using the LSD test. For data with heterogeneous variances, the Games-Howell test was applied. Non-normally distributed data were analyzed using nonparametric tests, with *post hoc* comparisons conducted via the Kruskal-Wallis test. A significance level of P ≤ 0.05 was considered statistically significant.

## 3 Results

### 3.1 The behavioral effects of taVNS on LPS-induced depression rats

**Figure 3:**
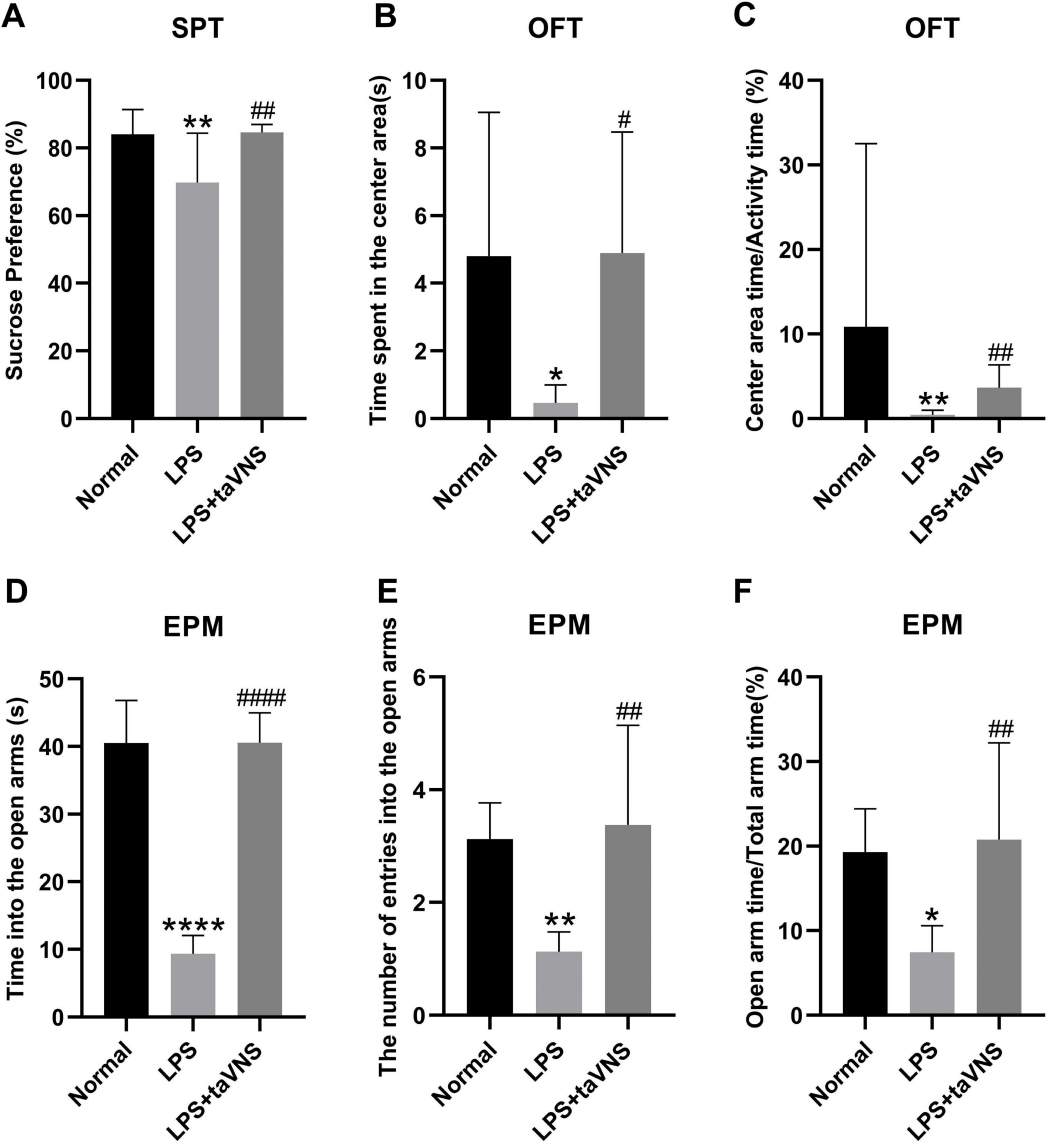
Comparison of depressive-like behaviors among different groups of rats. Data are presented as mean ± standard deviation (SD), with n = 8 per group. **(A)** Sucrose preference percentage for each group of rats. **(B)** Time spent in the center area in the open field test for each group of rats. **(C)** Ratio of center area time to activity time in the open field test for each group of rats. **(D)** Time spent in the open arms of the elevated plus maze for each group of rats. **(E)** The number of entries into the open arms of the elevated plus maze for each group of rats. **(F)** Ratio of open arm time to total arm time of the elevated plus maze for each group of rats.*P < 0.05,**P < 0.01, ****P < 0.0001 compared with the normal group; #P < 0.05, ##P < 0.01, ####P < 0.0001 compared with the model group.

In the sucrose preference test, the sucrose preference percentage was significantly lower in the model group compared with the normal group (P < 0.01). In contrast, the taVNS group exhibited a significant increase in sucrose preference percentage compared with the model group (P < 0.01) (**Figure 3A**).

In the open field test, the model group showed a significant reduction in time spent in the center area (P <0.05) and center area time/total activity time(P<0.01) compared with the normal group. Conversely, the taVNS group displayed a significant increase in both time spent in the center area (P < 0.05) and center area time/total activity time(P<0.01) compared with the model group (**Figures 3B, C**).

Similarly, in the elevated plus maze test, the model group demonstrated significantly decreased in the time spent in the open arms(P<0.0001) and the numbers of entries into the open arms (P < 0.01), and a significantly reduced ratio of open arm time to total arm time (P < 0.05) compared with that of the normal group. Compared to the model group, the taVNS group exhibited a significant increase in time spent in the open arms(P<0.0001), the numbers of entries into the open arms and the ratio of open arm time to total arm time (P < 0.01), (**Figures 3D–F**).

These behavioral findings suggest that taVNS intervention significantly alleviates LPS-induced depressive-like behaviors in rats.

### 3.2 Effects of taVNS on serum inflammatory cytokines in rats

**Figure 4:**
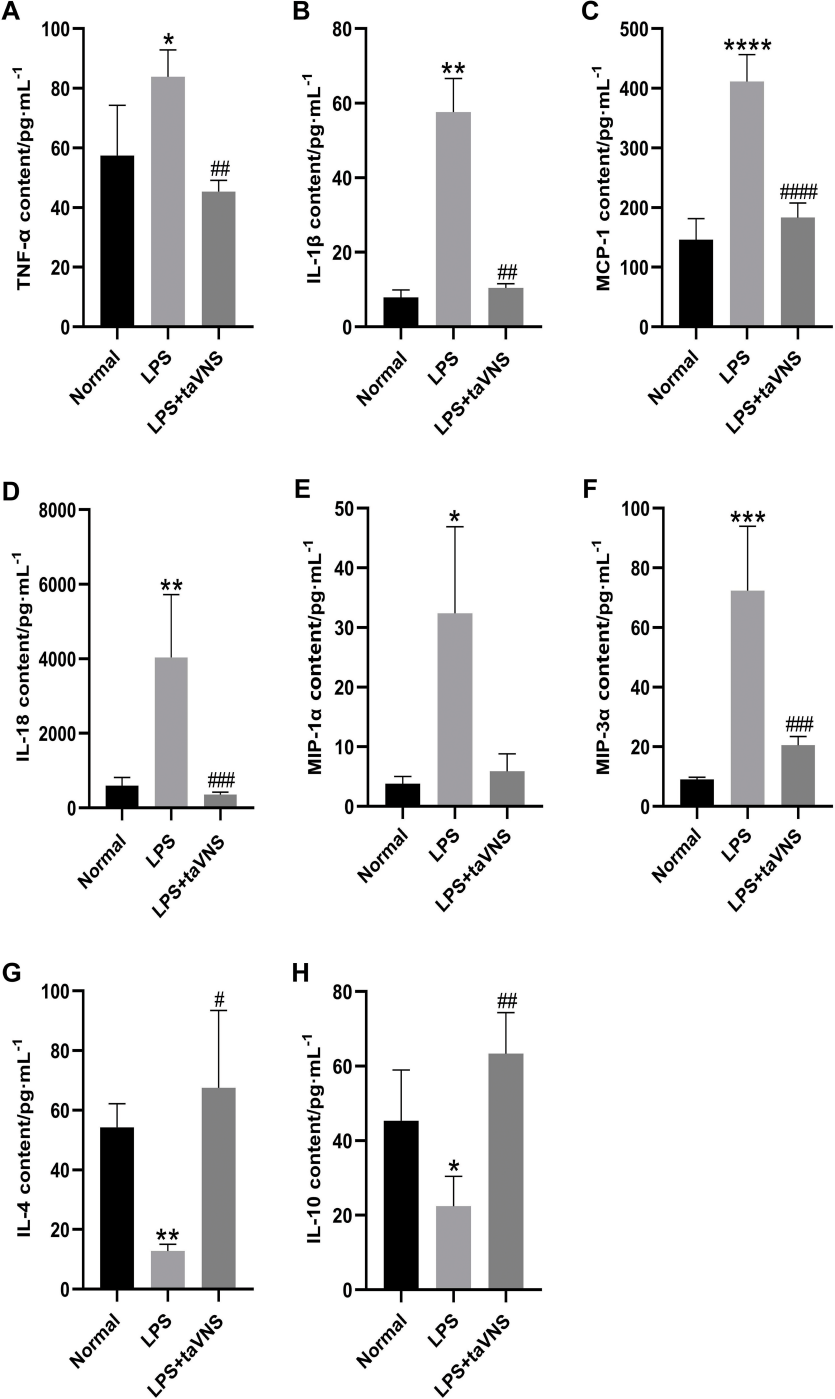
Comparison of inflammatory cytokine levels in the serum of rats across groups. Data are presented as mean ± standard deviation (SD), with n = 4 per group. **(A)** Serum TNF-α levels in each group. **(B)** Serum IL-1β levels in each group. **(C)** Serum MCP-1 levels in each group. **(D)** Serum IL-18 levels in each group. **(E)** Serum MIP-1α levels in each group. **(F)** Serum MIP-3α levels in each group. **(G)** Serum IL-4 levels in each group. **(H)** Serum IL-10 levels in each group.*P < 0.05, **P < 0.01, ***P < 0.001, ****P < 0.0001 compared with the normal group; #P < 0.05, ##P < 0.01, ###P < 0.001, ####P < 0.0001 compared with the model group.

Compared with the normal group, the serum levels of the anti-inflammatory cytokines IL-4 (P < 0.01) and IL-10 (P < 0.05) were significantly decreased in the model group (**Figures 4G, H**). In contrast, the levels of pro-inflammatory cytokines TNF-α (P < 0.05), IL-1β (P < 0.01), MCP-1 (P < 0.0001), IL-18 (P < 0.01), MIP-1α (P < 0.05), and MIP-3α (P < 0.001) were markedly elevated (**Figures 4A–F**).

Following taVNS intervention, the serum levels of IL-4 (P < 0.05) and IL-10 (P < 0.01) increased significantly (**Figures 4G, H**), while the levels of IL-1β (P < 0.01), MCP-1 (P < 0.0001), IL-18 (P < 0.001), and MIP-3α (P < 0.001) were significantly reduced compared with the model group. Although the serum levels of MIP-1α showed a downward trend in the taVNS group, the change did not reach statistical significance (P > 0.05) (**Figures 4A–F**).

These findings suggest that taVNS facilitates the production of anti-inflammatory cytokines and attenuates the excessive elevation of specific pro-inflammatory cytokines, highlighting its potential in regulating systemic inflammation in LPS-induced depressive-like states.

### 3.3 Effects of taVNS on TNF-α and NF-κB signaling pathway proteins in the prefrontal cortex

**Figure 5:**
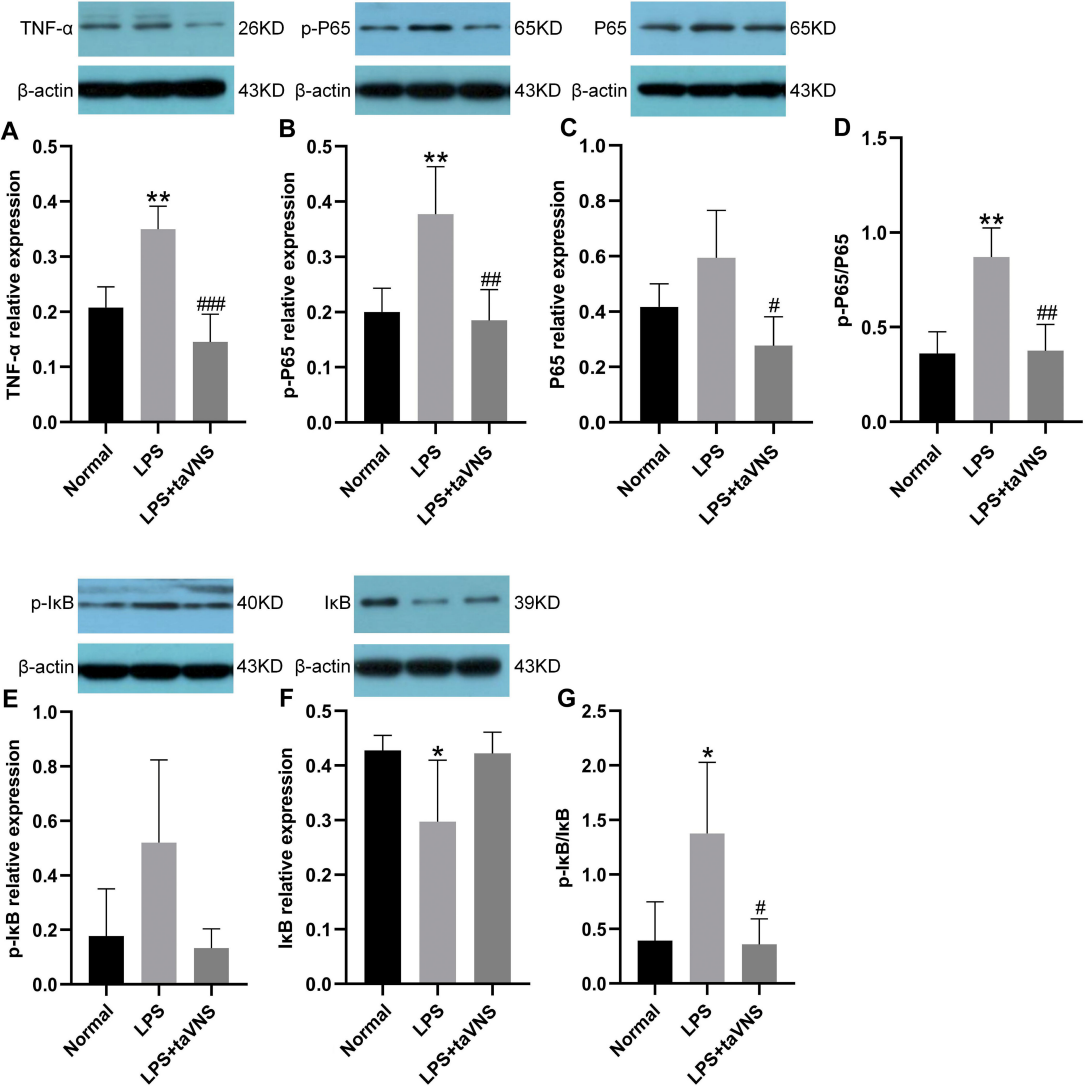
Comparison of inflammation-related protein levels in the prefrontal cortex across groups. Data are presented as mean ± standard deviation (SD), with n = 4 per group. **(A)** Relative expression level of TNF-α in the prefrontal cortex of each group. **(B)** Relative expression level of p-P65 in the prefrontal cortex of each group. **(C)** Relative expression level of P65 in the prefrontal cortex of each group. **(D)** Ratio of p-P65 to P65 in the prefrontal cortex of each group. **(E)** Relative expression level of p-IκB in the prefrontal cortex of each group. **(F)** Relative expression level of IκB in the prefrontal cortex of each group. **(G)** Ratio of p-IκB to IκB in the prefrontal cortex of each group.*P < 0.05, **P < 0.01 compared with the normal group; #P < 0.05, ##P < 0.01, ###P < 0.001 compared with the model group.

Compared with the normal group, the relative expression of the pro-inflammatory cytokine TNF-α protein in the prefrontal cortex was significantly increased in the model group after LPS induction (P < 0.01). Following taVNS intervention, the relative expression of TNF-α protein was significantly reduced compared with the model group (P < 0.001) (**Figure 5A**).

For NF-κB signaling pathway-related proteins, the expression of p-P65 protein in the model group was significantly elevated (P < 0.01), with a trend toward increased total P65 protein levels (P > 0.05), and the p-P65/P65 ratio was significantly higher (P < 0.01). In contrast, taVNS intervention led to a significant reduction in p-P65 protein expression (P < 0.01), a significant decrease in total P65 protein levels (P < 0.05), and a significantly lower p-P65/P65 ratio (P < 0.01) compared with the model group (**Figures 5B–D**).

Regarding IκB-related proteins, the model group showed a slight increase in p-IκB protein expression compared with the normal group (P > 0.05), a significant reduction in IκB protein levels (P < 0.05), and a significant increase in the p-IκB/IκB ratio (P < 0.05). Following taVNS intervention, the expression of p-IκB protein in the taVNS group exhibited a downward trend (P > 0.05), while IκB protein levels displayed an upward trend (P > 0.05), resulting in a significantly reduced p-IκB/IκB ratio (P < 0.05) compared with the model group (**Figures 5E–G**).

These findings indicate that taVNS regulates the expression of TNF-α and key NF-κB signaling pathway proteins in the prefrontal cortex, suppressing excessive activation of inflammation-related signaling. Specifically, taVNS reduces the phosphorylation levels of P65 and IκB and decreases TNF-α protein expression, thereby disrupting the positive feedback loop between TNF-α and NF-κB.

## 4 Discussion

Depression is widely recognized as a complex multisystem disorder closely associated with inflammation. In recent years, the roles of pro-inflammatory cytokines (e.g., TNF-α, IL-1β) and anti-inflammatory cytokines (e.g., IL-4, IL-10) in the pathophysiology of depression have been increasingly clarified. The sustained release of large amounts of stress- and inflammation-related mediators can trigger severe chronic neuroinflammation, characterized by elevated levels of central and peripheral inflammatory factors, which further exacerbate depressive symptoms (21). LPS has been shown to disrupt the permeability of the blood-brain barrier (BBB) in animal models, inducing neuroinflammation and oxidative stress (22), thereby increasing the levels of peripheral and central inflammatory cytokines and leading to depressive-like behaviors.

Our findings also demonstrated that LPS treatment in rats resulted in a significant reduction in sucrose preference percentage, decreased time spent in the central area and central area time compared to total activity time in the open field test, reduced time spent in the open arms, entries into the open arms in the elevated plus maze test, and a lower open-to-closed arm time ratio. These behavioral changes are indicative of depressive-like behaviors.

In recent years, both clinical and animal studies have shown that taVNS can effectively alleviate depressive-like behaviors (14, 23). Consistent with these findings, our study demonstrated that taVNS ameliorated LPS-induced depressive-like behaviors, as evidenced by increased sucrose preference, longer time spent in the central area and greater central area time compared to total activity time in the open field test, as well as increased exploratory time in the open arms of the elevated plus maze.These changes in behavior are closely related to changes in inflammatory cytokines.

IL-1β, a classical pro-inflammatory cytokine, exerts its effects by binding to IL-1 receptors, activating downstream NF-κB and MAPK signaling pathways, and amplifying both local and systemic inflammatory responses (24, 25). Numerous studies have demonstrated that elevated IL-1β levels are closely correlated with the severity of depressive symptoms. Beckett et al. reported that patients with higher IL-1β levels were more likely to exhibit resistance to antidepressant treatments, suggesting that IL-1β may serve as a predictive marker for therapeutic responses in depression (26).IL-18, another pro-inflammatory cytokine belonging to the IL-1 family, is often regarded as a key molecule acting synergistically with IL-1β in studies of depression and neuroinflammation (27, 28).In the present study, LPS treatment significantly increased serum IL-1β and IL-18 levels in rats, whereas taVNS intervention markedly reduced its expression. IL-1β and IL-18 can cross the blood-brain barrier (BBB) via a saturable transport mechanism, activating the central nervous system, particularly microglial cells in the hippocampus, thereby exacerbating localized neuroinflammation (29, 30). Additional studies have shown that inhibition of the IL-1β signaling pathway significantly reduces hippocampal inflammation and alleviates depression-like behaviors (31, 32).Our previous research has demonstrated that taVNS exerts anti-inflammatory and antidepressant effects by suppressing hippocampal IL-1β expression through the α7nAchR/NF-κB signaling pathway (14).It is suggested that taVNS may improve depressive symptoms by reducing peripheral IL-1β and IL-18 levels, thereby limiting its propagation to the central nervous system and subsequent activation of neuroinflammation.

Similar to IL-1β and IL-18, monocyte chemoattractant protein-1 (MCP-1) plays a critical role in immune cell migration and the propagation of inflammation. By binding to its receptor CCR2, MCP-1 promotes the migration of monocytes and macrophages to sites of inflammation. Additionally, it increases blood-brain barrier (BBB) permeability, facilitating the entry of peripheral immune cells into the central nervous system and amplifying the neuroinflammatory cascade (33, 34). It has been hypothesized that elevated MCP-1 levels not only enhance peripheral immune cell infiltration into the central nervous system but also activate microglial cells, triggering neuroinflammation and exacerbating depression-like behaviors (35, 36).

In this study, LPS treatment significantly increased MCP-1 levels, whereas taVNS intervention effectively reduced MCP-1 expression in serum. This reduction may alleviate microglial overactivation and localized inflammation by limiting the migration of peripheral immune cells to the central nervous system. Similar to pro-inflammatory factors such as IL-1β and MCP-1, MIP-1α and MIP-3α are key chemokines involved in immune cell recruitment and the propagation of inflammation, playing crucial roles in neuroinflammation and depression. Our study demonstrated that LPS treatment led to significant elevations in serum MIP-1α and MIP-3α levels in rats, while taVNS intervention reverses this expression.

In contrast to pro-inflammatory factors, anti-inflammatory cytokines are essential for maintaining immune system balance. IL-4 and IL-10 are classical anti-inflammatory cytokines that play complementary roles in modulating immune responses and mitigating inflammation.IL-4 promotes M2 macrophage polarization, inhibits the release of pro-inflammatory cytokines, and facilitates tissue repair (37, 38). Furthermore, IL-4 can modulate microglial activity through cytokine-cytokine receptor interactions, mitigating brain damage caused by neuroinflammation (39, 40). IL-10, another key anti-inflammatory cytokine, suppresses the production of pro-inflammatory mediators such as TNF-α and IL-1β, while enhancing tissue regeneration and immune homeostasis (41, 42).In depression-related studies, elevated IL-4 levels have been associated with better emotional stability (43). Moreover, decreased IL-10 levels have been observed in patients with major depressive disorder (44), and antidepressant treatments have been shown to increase IL-10 levels (43, 45).In this study, LPS treatment significantly decreased serum IL-4 and IL-10 levels in rats, whereas taVNS intervention markedly increased IL-4 and IL-10 levels. These findings suggest that taVNS may alleviate systemic inflammation and indirectly reduce central inflammation activation by enhancing anti-inflammatory cytokine levels. Our previous study also showed that taVNS may regulate the α7nAChR/JAK2/STAT3 signaling pathway in the spleen, reducing pro-inflammatory cytokines while enhancing the expression of anti-inflammatory cytokines, thereby alleviating systemic inflammatory burden (16).

This study further elucidates the regulatory effects of taVNS on the NF-κB signaling pathway. NF-κB is a central regulatory factor in neuroinflammation, and its activation is often accompanied by upregulation of pro-inflammatory cytokines and disruption of synaptic function (46, 47). TNF-α is a critical downstream effector of the NF-κB signaling pathway and also a key participant in its positive feedback regulation (48).The interplay between central and peripheral TNF-α is particularly significant in inflammatory responses and neuropathological processes (48). Additionally, TNF-α released peripherally can penetrate the blood-brain barrier, influencing central neuroinflammation, and may further activate the NF-κB signaling pathway in neurons and glial cells, thereby creating a vicious cycle of inflammation (49). This interplay between central and peripheral NF-κB/TNF-α signaling is considered a core mechanism underlying inflammation-induced depression-like behaviors. Kang et al. demonstrated in an LPS model that activation of the NF-κB signaling pathway is closely associated with increased levels of TNF-α, which exacerbate neuroinflammation and induce depression-like behaviors (50, 51).

Phosphorylated p65 (p-p65) is a key marker of NF-κB activation. It is released into the nucleus following IκB degradation, where it binds to DNA and initiates the expression of pro-inflammatory cytokines (52).

IκB (inhibitory κB protein), the main negative regulator of NF-κB, typically forms a complex with NF-κB under normal conditions, preventing its nuclear translocation (53). Upon stimulation by LPS or similar factors, IκB is rapidly phosphorylated by IκB kinase (IKK), leading to its degradation and subsequent activation and nuclear translocation of the NF-κB/p65 subunit (54). Studies have shown that LPS-induced IκB phosphorylation and degradation, along with p65 phosphorylation, not only amplify peripheral and central inflammatory responses but also induce excessive expression of pro-inflammatory cytokines, further activating microglial inflammation and causing neuronal damage (55).

Our pervious study showed that the expression of TNF-α significantly increased and the expression of p-Jak2 and p-STAT3 markedly decreased in the hypothalamus, amygdala (15) and hippocampus (56) induced by LPS. In the present study, LPS treatment significantly increased the expression of TNF-α, p-p65 levels and the p-p65/p65 ratio, while reducing IκB stability in the prefrontal cortex. Additionally, it elevated serum TNF-α levels, indicating robust activation of the NF-κB pathway. TaVNS intervention markedly reduced TNF-α expression, p-p65 levels and the p-p65/p65 ratio, while enhancing IκB stability in the prefrontal cortex. TaVNS also increased the expression of TNF-α in the hypothalamus and amygdala (15). It also decreased peripheral TNF-α levels. These findings suggest that taVNS may mitigate neuronal damage and improve depression-like behaviors by inhibiting excessive activation of the NF-κB/TNF-α pathway in both the prefrontal cortex and peripheral systems.

Microglia are critical nervous system-specific immune cells. LPS administration significantly increased the density of IBA-1+microglia in the prefrontal cortex (57). So the increased inflammatory expression in the prefrontal cortex may be mediated by activated microglia. Furthermore, our previous results indicate that the anti-inflammatory effects of taVNS in the prefrontal cortex may involve other signaling pathways on the microglia. For instance, taVNS may exert its antidepressant effects through the purinergic P2X7 receptor/NLRP3 inflammasome/IL-1β signaling pathway and the TLR4/MyD88 pathway in the prefrontal cortex (58, 59). In our present study, taVNS exhibited the anti-inflammatory and antidepressant effects for acute inflammation induced by LPS may be through the NF-κB/TNF-α pathway on microglia in the prefrontal cortex. Previous studies have shown that taVNS can have a regulatory effect on norepinephrine (NE) (60), and NE has been shown to modulate immune cell responses leading to increased anti-inflammatory and blunting of pro-inflammatory effects (61). Therefore, taVNS may have an anti-inflammatory effect on the upstream brain region including PFC through microglia and its signaling pathway by regulating NE, and thus generate anti-depression. This may be one of our future research directions.

While this study offers new insights into the anti-inflammatory and antidepressant mechanisms of taVNS, several limitations should be acknowledged. The study primarily focused on overall changes in inflammatory signaling without distinguishing the specific contributions of neurons, microglia, and astrocytes in inflammation regulation. We will continue to explore the mechanism among neurons, microglia, and astrocytes in depth in future studies.

## 5 Conclusions

In conclusion, this study demonstrates that taVNS significantly improves LPS-induced depression-like behaviors by modulating peripheral pro-inflammatory cytokines (e.g., IL-1β and TNF-α) and anti-inflammatory cytokines (e.g., IL-4,IL-10) and inhibiting the activation of NF-κB signaling pathway in the prefrontal cortex. It is suggested that taVNS exerted antidepressant effects by regulating peripheral pro-inflammatory factors and central neuroinflammation, highlighting its critical role in the peripheral-central inflammation regulation network. These findings provide critical theoretical evidence for understanding the pathophysiology of inflammation-related depression and for exploring taVNS-based non-invasive neuroregulatory interventions.

## Data Availability

The original contributions presented in the study are included in the article/**Supplementary Material**, further inquiries can be directed to the corresponding authors.
