# Supplementary figures and images for: Transcutaneous auricular vagus nerve stimulation alleviates inflammation-induced depression by modulating peripheral-central inflammatory cytokines and the NF-κB pathway in rats
Source: Front Immunol. 2025 May 16;16:1536056. doi: 10.3389/fimmu.2025.1536056 (PMC12122300; doi:10.3389/fimmu.2025.1536056)

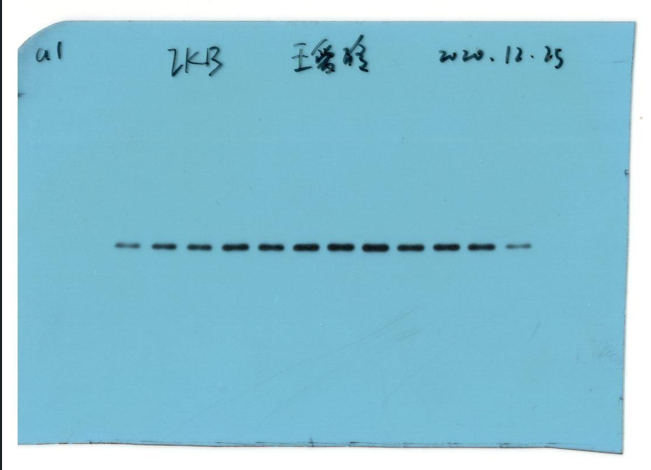

Supplement: Supplementary file 1 [file Image1.png]

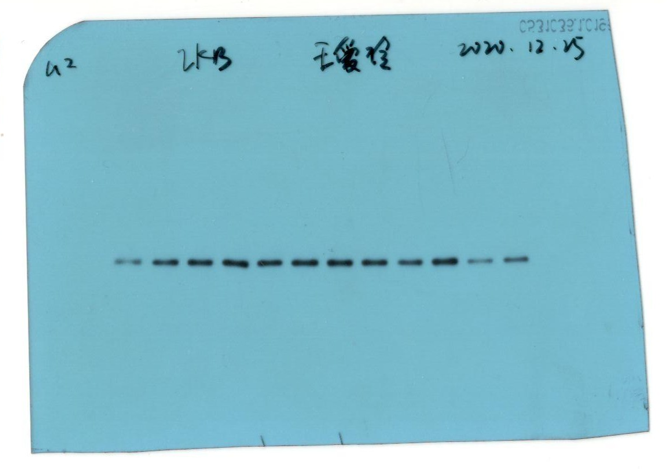

Supplement: Supplementary file 2 [file Image2.png]

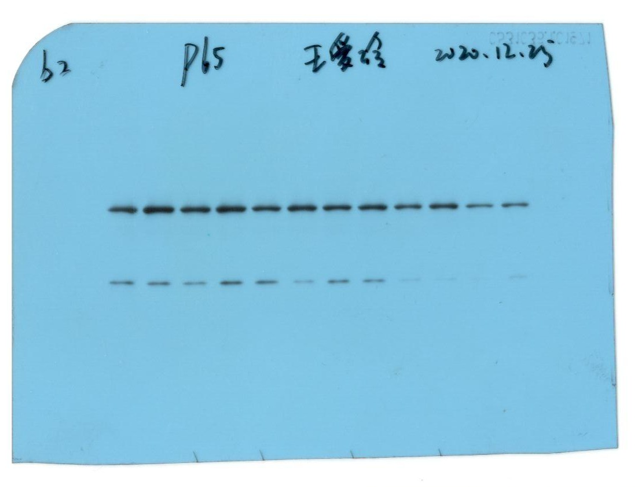

Supplement: Supplementary file 3 [file Image3.png]

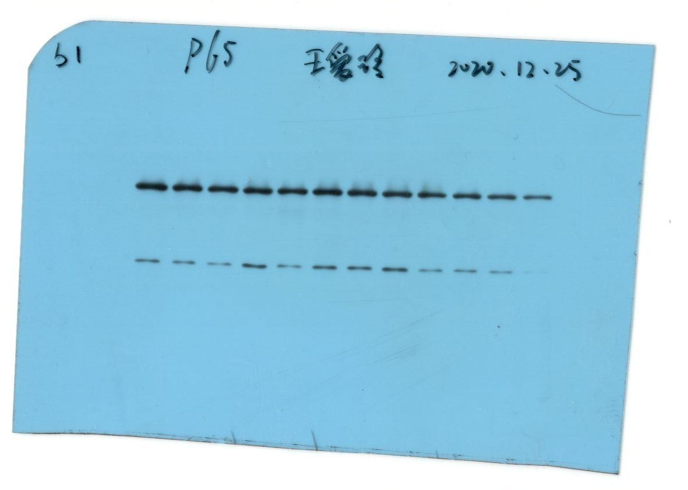

Supplement: Supplementary file 4 [file Image4.png]

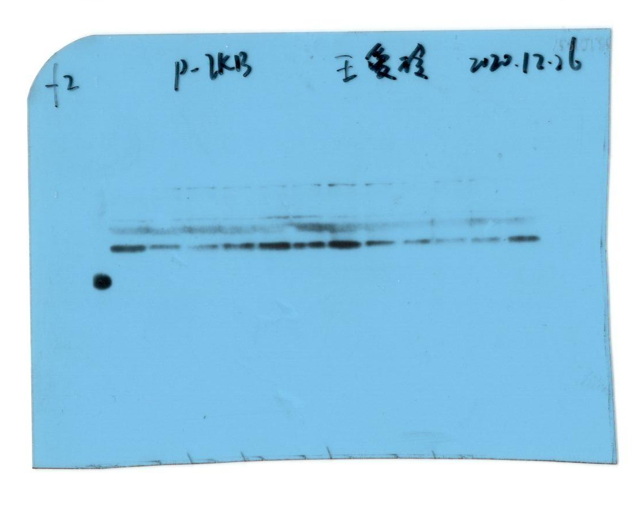

Supplement: Supplementary file 5 [file Image5.png]

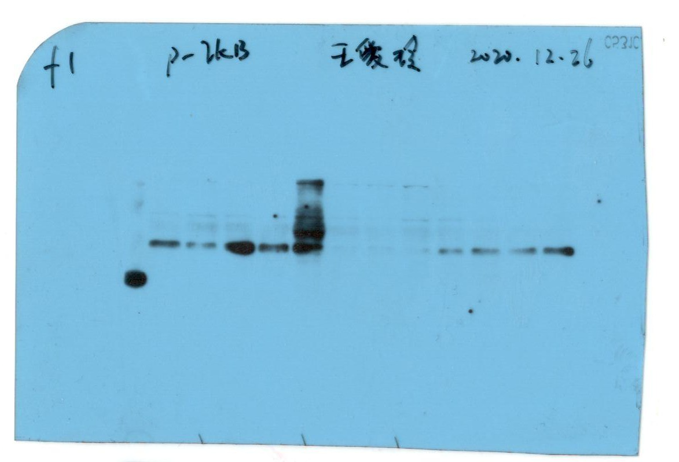

Supplement: Supplementary file 6 [file Image6.png]

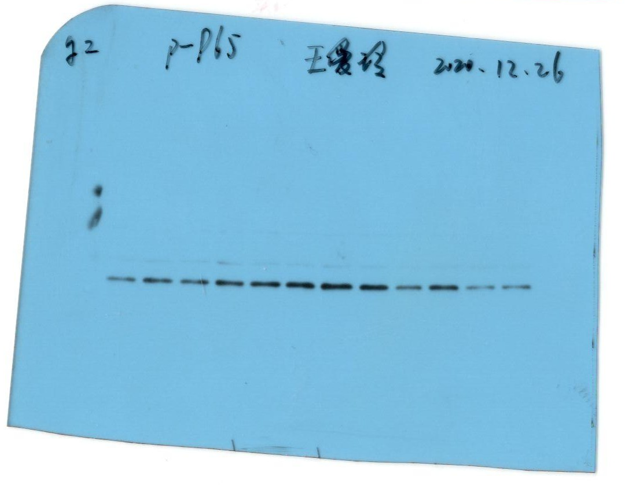

Supplement: Supplementary file 7 [file Image7.png]

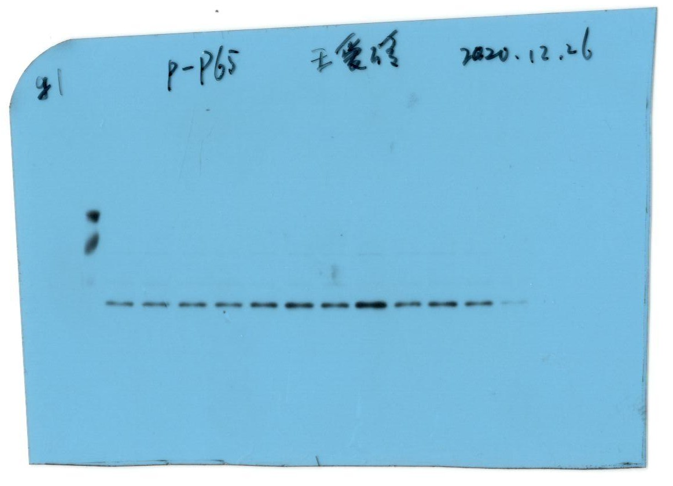

Supplement: Supplementary file 8 [file Image8.png]

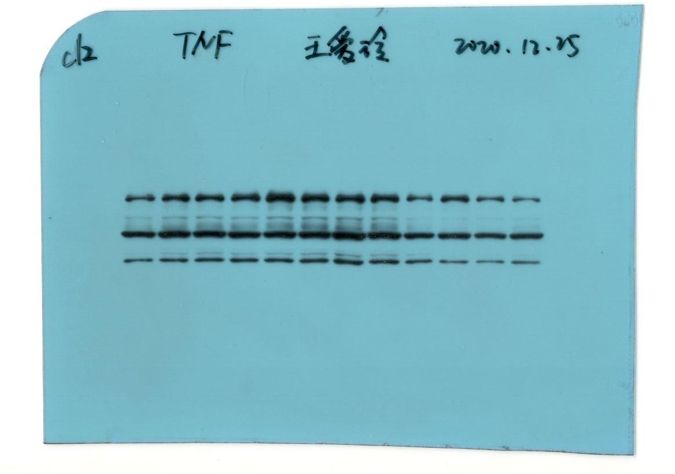

Supplement: Supplementary file 9 [file Image9.png]

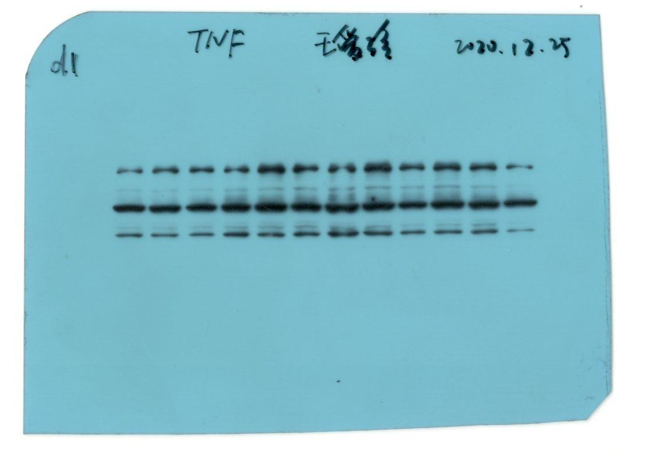

Supplement: Supplementary file 10 [file Image10.png]
